# Adrenocortical Carcinoma: Complete Surgical Resection After 18 Years

**DOI:** 10.4021/wjon369w

**Published:** 2011-12-19

**Authors:** Harsha V. Polavarapu, Sergio Casillas, Pamela Edmonds, Philip S. Lim, Christopher M. Pezzi

**Affiliations:** a Departments of Surgery, Abington Memorial Hospital, Abington, PA, USA; b Departments of Pathology, Abington Memorial Hospital, Abington, PA, USA; c Departments of Radiology, Abington Memorial Hospital, Abington, PA, USA

**Keywords:** Adrenocortical carcinoma, Weiss criteria

## Abstract

Adrenal cortical carcinoma (ACC) is a rare neoplasm often associated with an aggressive biological behavior. Complete surgical resection is the mainstay of therapy for ACC and offers the best chance for prolonged disease-free survival. We present an unusual case of a long-standing adrenal mass, well documented over a period of at least 18 years, without the development of metastatic disease, and ultimately proven to represent ACC after successful surgical resection. Physicians should be aware that ACC can present with a wide spectrum of biological behavior, from very aggressive to more indolent disease.

## Introduction

Adrenal cortical carcinoma (ACC) is a rare neoplasm often associated with an aggressive natural history. As the second most aggressive endocrine malignancy after anaplastic carcinoma of thyroid, ACC accounts for approximately 0.2% of all cancer deaths in the United States annually, with an incidence of approximately 1 to 2 cases per million people per year [1-4]. We present an unusual case of a long-standing adrenal mass, well documented over a period of at least 18 years, without the development of metastatic disease, which was ultimately proven to represent ACC after successful surgical resection.

## Case Report

A 47-year-old woman was referred by her primary physician for a surgical opinion concerning an asymptomatic, 14 cm, right adrenal mass described on recent magnetic resonance imaging (MRI). The right adrenal mass had been previously radiographically identified at least as far back as 1992. Surgical resection had been recommended to the patient back in 1992 at another institution, but she had declined, reportedly because of a fear of the major surgery at the time. Therefore, the mass was simply followed intermittently over the ensuing 18 years at irregular intervals without a definitive diagnosis or any treatment. Her past medical history included attention deficit disorder, asthma, benign cystic breast disease, breast reduction surgery and a recently identified 9 x 4.6 cm left adnexal mass. On careful questioning, she denied any symptoms from the large adrenal mass, including any pain, fullness, nausea, vomiting, dyspepsia or increasing abdominal girth. Abdominal examination was also normal, without any palpable mass or other related findings.

**Figure 1:**
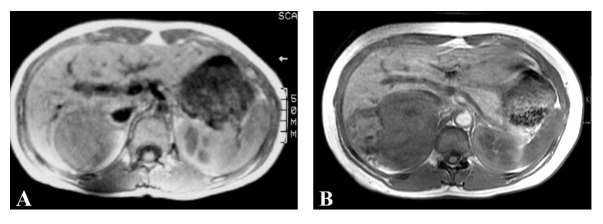
Serial MRI examinations spanning 18 years, showing low signal mass in the right adrenal gland which is irregular and heterogeneous on (A) Axial T1 spin echo measuring 6 cm in 1992 and (B) Axial T1 inphase gradient echo sequence measuring 14 cm in 2010.

Laboratory studies revealed a serum cortisol level of 10.7 mcg/dL (normal 3-17), serum aldosterone of 2 ng/dL (normal 3-16), plasma renin of 1.46 ng/mL/h (normal 0.25-5.82) and serum metanephrines of 131 pg/mL (normal < 205) indicating that the tumor was non-functioning. MRI of the abdomen revealed a 14 cm, irregular, right adrenal mass with heterogeneous density and attenuation characteristics concerning for malignancy. Comparison with a previous MRI examination provided by the patient from 1992 revealed that the mass had slowly increased in size (from 6 cm to 14 cm) and changed in appearance over the period of 18 years (Fig. 1A, B). There was no evidence of metastatic disease seen to any lymph nodes, the liver, or the lungs.

Surgical resection of the large, and enlarging, right adrenal mass was recommended both for diagnosis and for treatment. However, the patient first underwent a laparoscopic left salpingo-oophorectomy to exclude malignancy in the large, complex, left adnexal mass. Final pathology from the adnexal mass was a benign luteal cyst, hydrosalpinx, and endometriosis. After successful recovery from the salpingo-oophorectomy, elective right adrenalectomy was undertaken using an open, trans-abdominal approach through a generous right subcostal incision.

At laparotomy, the large adrenal mass was found adherent to the capsule of the liver posteriorly and inseparable from the right diaphragm. It did not involve the right kidney. A separate small mass was also palpable in the fundus of the gallbladder, distant from the adrenal mass. An en-bloc resection of the entire adrenal mass, along with a portion of the right diaphragm and a portion of the capsule of the right lobe of the liver was performed. The diaphragm was closed primarily with permanent sutures without a chest tube. Simple cholecystectomy was also performed, with a benign intraoperative frozen section of the gallbladder mass. The estimated blood loss was 100 cc, and she received 2 units of packed red blood cell transfusion intraoperatively.

The postoperative course was initially unremarkable. The patient was discharged home on postoperative day 4. She was however readmitted 2 weeks postoperatively with shortness of breath secondary to a large, right-sided, pleural effusion. The pleural effusion required drainage using a temporary pigtail catheter. She was again discharged, and then fully recovered.

**Figure 2:**
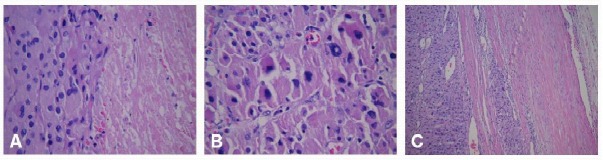
Hematoxylin and eosin staining of the tumor demonstrating 3 of the Weiss criteria of ACC: (A) tumor necrosis, 400x; (B) grade IV nuclei, 400x; (C) invasion into, but not through the capsule, 100x.

The final pathology revealed the adrenal tumor to be 14 cm in maximum diameter, with a weight of 566 gm. Extensive necrosis was present, along with gross calcifications. The tumor was densely adherent and fibrosed to the diaphragm, but not actually invading microscopically. Sections showed a neoplastic process consisting of large polygonal cells with abundant granular eosinophilic cytoplasm. Nuclei were markedly variable in size, with some showing large intra-nuclear inclusions. Extensive zonal necrosis was present. Invasion into, but not through, the capsule was present. The nuclear grade was high (Fuhrmann grade IV). The tumor was oncocytic with less than 25% of clear cells. There was a diffuse growth pattern (Fig. 2A, B, C). Based on a Weiss Score of 5 (of 9) the final pathologic diagnosis was adrenocortical carcinoma. Additional microscopic features that did not meet the Weiss criteria were as follows: the tumor did not exceed a mitotic rate of more than 5/50 high power fields, there were no atypical mitoses, the tumor was not sinusoidal, and there was no vascular invasion identified.

No adjuvant therapy was recommended. The patient continues to do well and no evidence of disease 7 months after the surgical resection.

## Discussion

ACCs are frequently large, and size has been reported to be a prognostic factor [1]. A very large ACC is concerning for a late or terminal stage in the natural history of the disease. However, despite the presence of a 14 cm tumor, and its existence for at least 18 years, the patient reported had no evidence of distant metastasis and was ultimately still surgically resectable.

Although ACCs can develop at any age, there is a bimodal age distribution, with disease peaks before the age of 5 years and in the fourth to fifth decade of life. In general, the level of aggressiveness and pace of disease progression are more rapid in adults than in children [5].

Nearly half of ACCs are functional, frequently resulting in symptoms that lead to recognition and diagnosis. Many of the nonfunctional tumors will remain without signs or symptoms of the disease until the tumor either becomes large enough to cause symptoms from compression of nearby structures, or the patient develops symptoms from the metastatic disease. The remainder is identified incidentally on radiographic imaging done for other reasons. The patient reported was symptom-free and the mass was non-functional. Her tumor was identified incidentally on radiographic imaging.

Computerized tomography (CT) is useful in distinguishing benign adrenal adenomas from ACC. MRI is a complementary diagnostic modality to CT, in that local invasion and involvement of the vena cava are more readily identifiable. Imaging phenotype consistent with ACC include the following: irregular shape; large size (> 4 cm in diameter); intralesional calcification; tumor heterogeneity on both plain films and contrast enhancement, which may indicate intralesional hemorrhage, necrosis, or both; unilateral location; high CT attenuation values (especially with > 20 HU) and evidence of tumor invasion of local structures or extension into major vessels [6]. Imaging of our patient showed many of the typical characteristics of ACC, with a large, unilateral, irregular, partially calcified, heterogenous mass. However invasion of local structures or vascular invasion was not seen.

Despite multiple improvements in the diagnosis and treatment of other types of solid malignancies over the past 2 decades, ACC continues to be a highly lethal malignancy with poor overall survival and few effective treatment options [7]. Complete surgical resection is the mainstay of therapy for ACC and offers the best chance for prolonged disease-free survival and cure. The principle prognostic factors for ACC are complete surgical resection of the tumor and an early stage at diagnosis [8]. In our patient there was microscopic invasion of the tumor into, but not through, the tumor capsule. The portion of the diaphragm that was removed represented adherence by a fibrotic reaction from the longstanding tumor, but was without invasive tumor involvement into the muscle on microscopic examination.

Weiss proposed a scoring system which consists of nine criteria to aid in the diagnosis of adrenal cancer [9]. The presence of three or more of the nine features from the Weiss criteria is highly suggestive of ACC [10]. Our patient’s tumor demonstrated high nuclear grade, capsular invasion, less than 25% clear cells, diffuse growth pattern, and tumor necrosis (5/9 criteria); leading to the classification of the mass as ACC.

Adjuvant treatment with mitotane has been shown to be beneficial in patients with ACC [11]. Given that an R0 resection was achieved, and because of the very long history suggesting a more indolent tumor, adjuvant therapy was not recommended in this case. Since the biological behavior of these tumors is often aggressive, and the development of local-regional recurrence and/or metastatic disease is not uncommon, hence long-term follow-up is important for all patients after treatment.

The existence of this malignant tumor for at least 18 years prior to resection is extremely unusual. To the best of our knowledge, this is the first case of ACC to be reported with a well-documented 18-year untreated interval prior to successful surgical resection.
